# Characterization and molecular docking study of cathepsin L inhibitory peptides (SnuCalCpIs) from *Calotropis procera* R. Br

**DOI:** 10.1038/s41598-022-09854-x

**Published:** 2022-04-06

**Authors:** Chang Woo Kwon, Subin Yeo, Pahn-Shick Chang

**Affiliations:** 1 Research Institute of Agriculture and Life Sciences, Seoul National University, Seoul, 08826 Republic of Korea; 2 Department of Agricultural Biotechnology, Seoul National University, Seoul, 08826 Republic of Korea; 3 Center for Food and Bioconvergence, Seoul National University, Seoul, 08826 Republic of Korea; 4 Center for Agricultural Microorganism and Enzyme, Seoul National University, Seoul, 08826 Republic of Korea

**Keywords:** Cancer prevention, Peptides, Proteins, Proteases

## Abstract

Propeptides, released from the autocatalytic activation of its zymogen, are potential inhibitors against proteases involved in cancer cell invasion and migration. Our research team previously obtained novel propeptides (SnuCalCpIs) from transcriptome analysis of the medicinal plant *Calotropis procera* R. Br. and reported them as promising candidates for cancer therapeutics due to their cathepsin L inhibition activity. In the present study, inhibitory activity among SnuCalCpIs was compared with inhibition efficiency and verified by *in silico* molecular docking analysis. Only SnuCalCpI03 and SnuCalCpI15, expressed in *Escherichia coli*, showed inhibitory activity against cathepsin L as competitive inhibitors, and the half-maximal inhibitory concentrations (IC_50_) values of 2.1 nM and 1.6 nM, respectively. They were stable below 70 °C, maintaining more than 90% inhibitory activity over a wide range of pH (2.0–10.0), except at the isoelectric point (pI). The template-based docking simulation models showed that SnuCalCpI02, SnuCalCpI12, and SnuCalCpI16 could not interact with the substrate-binding cleft of cathepsin L even though they possessed the same conserved domain. In contrast, SnuCalCpI03 and SnuCalCpI15 interacted with cathepsin L along the propeptide binding loop and substrate-binding cleft, resulting in obstruction of substrate access to the active site.

## Introduction

Proteolytic enzymes (also termed proteases, proteinases, and peptidases) hydrolyze peptide bonds in proteins and are critical for malignant tumor cell progression^1^. Lysosomal proteases such as cathepsins have emerged as potential effectors that change the tumor microenvironment by degrading various growth factors (chemokines and cytokines) and the extracellular matrix (ECM)^2,3^. Cathepsins are also involved in the detachment of cell–cell adhesion molecules and contribute to tissue invasion, and therefore represent a crucial class of proteases that promote cancer progression and metastasis^4^. Although many cathepsins have no effect or a tumor-promoting function in specific cancer types, cathepsin L has a unique ability to either suppress or enhance carcinogenesis in a context-dependent manner. Cathepsin L is often expressed at high levels in tumors^5^. In some instances, particularly in breast, pancreatic, and head and neck cancer, cathepsin L expression is a valuable biomarker with a negative prognostic indicator for patients' time to relapse and overall survival^6–8^. Therefore, cathepsin L is regarded as an attractive target for inhibitor development as a potent therapeutic agent.

Various types of cathepsin L inhibitors have been investigated as cancer cures^9–11^. Among them, high-molecular-weight protein inhibitors have attracted interest as a potential alternative or adjunct therapy for the treatment of cancer invasion. Unlike chemical inhibitors that bind cysteine residues at the active site regardless of the type of enzyme, these protein inhibitors have higher enzyme selectivity resulting from stereospecificity of the protein–protein interaction occurring only at the specific enzyme surface. Cysteine protease propeptides, promising inhibitors released from the autocatalytic activation of its proenzyme, were obtained from *Calotropis procera* R. Br. and termed as SnuCalCpIs^12^. Among them, SnuCalCpI03 displayed remarkable inhibition of lung, colon, breast, pancreatic, and prostatic cancer cell invasion by interacting with cathepsin L secreted from cancer cells^13^. The propeptide chain bends along the propeptide binding loop of cathepsin L and covers the substrate-binding cleft, thereby blocking substrate access to the active site. However, the in-depth interaction between cathepsin L and SnuCalCpIs is not known.

A few protein–protein complex structures have been successfully determined and deposited in the Protein Data Bank (PDB) over the past few decades. However, the number of protein complexes in the PDB is still minimal compared with the number of individual protein molecules because of the high cost and technical difficulties in finding optimum conditions. As such, protein–protein docking simulation, which computationally predicts the interaction between two proteins, has played a crucial role in producing structural information for two different proteins and larger complexes^14,15^. Computational docking methods designed for determining interactions between more flexible and larger peptide molecules are more suitable for the modeling than small-molecule-proteins. Template-based methods, in particular, usually yield higher-quality predictions if good templates are available^16^.

In this study, we used six peptide inhibitors (SnuCalCpIs), obtained from *Calotropis procera* R. Br., which might inhibit cathepsin L activity, and compared their characteristics, e.g., inhibition efficiency, type, and stability. Furthermore, we used *in silico* molecular docking analysis to interpret the differences in inhibition activity between cathepsin L and SnuCalCpIs despite conserved domains in the SnuCalCpIs.

## Materials and methods

### Plant material

*Calotropis procera* R. Br., cultivated in Myanmar, was kindly gifted from 3 M Korea Ltd. (Seoul, South Korea) and identified by Dr. Byoung-Cheorl Kang at Seoul National University. It was grown in a greenhouse at the agricultural experiment farm of Seoul National University (Suwon, South Korea). The experimental research of the plant material used in this study complies with relevant institutional, national, and international guidelines and legislation.

### RNA extraction and cDNA synthesis

Total RNA was extracted from *Calotropis procera* R. Br. using an RNeasy® plant mini kit (Qiagen, Valencia, CA, USA). A young leaf of *Calotropis procera* R. Br. was ground in a liquid nitrogen medium, and RNA was extracted according to the manufacturer's protocol. RNA was eluted with 30 µL of RNase-free water, and the RNA concentration was quantified using a NanoDrop spectrophotometer at a wavelength of 260 nm. Samples with RNA integrity values above 8.3 determined by an Agilent 2100 Bioanalyzer (Agilent Technologies, Santa Clara, CA, USA) were used. cDNA was synthesized with 5 µg of RNA using a PrimeScript™ first-strand cDNA synthesis kit (Takara, Otsu, Japan). Synthesis of cDNA was carried out with RNase inhibitor, oligo dT primer, total RNA, 5X PrimeScript buffer, dNTP mixture, PrimeScript RTase and dNTP mixture.

### Amplification of SnuCalCpI genes and construction of recombinant plasmids

SnuCalCpI genes (GenBank: GBHG01000000 and GBZK01000000) were amplified using first-strand cDNA and gene-specific primers (Table S1), including restriction sites (*Nde*I and *Xho*I) and In-Fusion HD cloning sites. The first-strand cDNA was screened according to the sequence identity (> 40%) with cathepsin L propeptide. PCR amplification was carried out as follows: 95 °C for 2 min; 95 °C for 30 s, 53 °C for 30 s, 72 °C for 60 s (27 cycles); 72 °C for 5 min. Duplicate PCR products were purified from 1% agarose gel electrophoresis using a QIAEX II® Gel Extraction Kit (QIAGEN) and sequenced by an ABI 3730xl DNA analyzer. The purified PCR products were cloned with a linearized pET29b( +) vector, which was digested using *Nde*I and *Xho*I. The recombinant plasmid was then transformed into competent *Escherichia coli (E. coli*) DH5α cells and replicated, purified, and sequenced.

### Expression and purification of SnuCalCpIs

The purified recombinant plasmid from competent *E. coli* DH5α cells was transformed into *E. coli* BL21 (DE3) Star competent cells for protein expression. A single colony grown on an LB agar plate containing 50 μg/mL kanamycin was cultured overnight in 5 mL LB broth containing 50 μg/mL kanamycin at 37 °C with shaking. A 2 mL volume of the overnight culture was inoculated into 200 mL of fresh LB medium with 50 μg/mL kanamycin and incubated at 37 °C with shaking until the optical density at 600 nm (OD_600_) was between 0.6 and 0.8. Subsequently, expression of the SnuCalCpI genes was induced by adding 0.5 mM isopropyl-β-D-thiogalactopyranoside (IPTG) at 20 °C for 12 h. After centrifugation at 10,000xg for 10 min at 4 °C, the supernatant was decanted and the cell pellet resuspended in 50 mM Tris–HCl buffer (pH 8.0) with 300 mM NaCl and 10 mM imidazole. Sonication was carried out to disrupt the cell pellet with 10 cycles of 10 s pulse, 10 s pause, and 35% power. The cell lysates were separated into pellet and supernatant by centrifugation at 12,000xg for 20 min and used for SDS-PAGE analysis. The His-tagged SnuCalCpIs in the supernatant was purified by Ni–NTA chromatography. The Ni–NTA column was washed with the lysis buffer (10 mM imidazole, 300 mM NaCl, and 50 mM Tris–HCl, pH 8.0). Next, the supernatant was added to the Ni–NTA resin and gently mixed for 30 min at 4 °C. Unbound proteins were decanted, and the resin was washed with the washing buffer (20 mM imidazole, 300 mM NaCl, and 50 mM Tris–HCl, pH 8.0). The adsorbed proteins were eluted using elution buffer (250 mM imidazole, 300 mM NaCl, and 50 mM Tris–HCl, pH 8.0), and the eluent was dialyzed for inhibition assays.

### Determination of inhibitory activity against papain

The inhibitory activity of SnuCalCpIs against papain (Sigma Co., Missouri, USA) was determined by measuring the residual enzyme activity towards N-α-Benzoyl-DL-arginine-β-naphthylamide (BANA). The reaction mixture was prepared as follows: 3.6 µg/mL papain, 0–28.6 µg/mL SnuCalCpIs, 7.1 mM 2-mercaptoethanol, and 0.3 mM BANA in 71 mM sodium phosphate buffer, pH 6.0, containing 1.4 mM ethylenediaminetetraacetic acid (EDTA)^17^. The mixture was pre-incubated for 10 min at 37 °C without BANA. Then, the enzyme reaction was initiated by adding BANA dissolved in DMSO at 37 °C. The enzyme reaction was terminated by adding 1 mL of 0.06% (w/v) *p*-dimethylaminocinnamaldehyde/ethanol and 1 mL of 2% (v/v) HCl/ethanol. The reactant was kept at room temperature for 30 min for color development, and the intensity of β-naphthylamine was measured at 540 nm using a spectrophotometer. The relative residual activity of papain was evaluated using the following equation:

$$ {\text{Relative residual activity }}\left( \% \right) = \frac{{\text{T*}}}{{\text{T}}} \times 100 $$

where T denotes the OD_540 nm_ in the absence of SnuCalCpIs, and T* is the OD_540 nm_ in the presence of SnuCalCpIs.

### Determination of inhibitory activity against cathepsin L

The inhibitory activity of SnuCalCpIs against cathepsin L (BioVision Inc., California, USA) was determined by measuring the residual enzyme activity towards Z-Phe-Arg-7-amino-4-methylcoumarin hydrochloride (Z-Phe-Arg-AMC)^18^. The reaction mixture was prepared as follows: 0.2 nM cathepsin L, 0–9.5 nM SnuCalCpIs, 10 mM dithiothreitol, and 0.6 µM Z-Phe-Arg-AMC in 100 mM sodium phosphate buffer, pH 6.0, containing 2 mM EDTA. The mixture was pre-incubated for 10 min at 30 °C without Z-Phe-Arg-AMC. Then, the enzyme reaction was initiated by adding Z-Phe-Arg-AMC. The activity of cathepsin L was determined by measuring the fluorescent intensity of AMC with excitation/emission peak wavelengths at 355/460 nm in a fluorometer. The relative residual activity of cathepsin L was evaluated using the following equation:

$$ {\text{Relative residual activity }}\left( \% \right) = \frac{{\text{T*}}}{{\text{T}}} \times 100 $$

where T denotes the initial velocity in the absence of SnuCalCpIs and T* is that in the presence of SnuCalCpIs.

### Enzyme inhibition kinetics

The inhibition mode of SnuCalCpIs was determined from its inhibitory activity against cathepsin L. SnuCalCpIs (0, 20, and 40 nM) were mixed with cathepsin L, and the mixtures were pre-incubated for 10 min at 30 °C. Inhibitory activity was measured with the addition of each substrate at concentrations ranging from 0.5 to 2.0 µM. The kinetic parameters and the mode of enzyme inhibition were determined from Lineweaver–Burk plots and the initial velocities of cathepsin L activity^19^.

### Effects of temperature and pH

The effects of temperature and pH on the inhibitory activity were determined by comparing relative inhibitory activity against cathepsin L. The thermostability of the inhibitor samples was evaluated by measuring the residual inhibitory activity after incubation in temperatures ranging from 30 to 100 °C for 30 min in 50 mM Tris–HCl buffer (pH 8.0). For the determination of pH stability, inhibitor samples were incubated in 100 mM glycine–HCl buffer over a pH range of 2.0–3.0, 100 mM sodium acetate buffer over a pH range of 4.0–5.0, 100 mM sodium phosphate buffer at pH 6.0, 100 mM Tris–HCl buffer over a pH range of 7.0–8.0, and 100 mM glycine–NaOH buffer over a pH range of 9.0–10.0 at 4 °C for 24 h.

### Circular dichroism

Far-ultraviolet (UV) (190–260 nm) circular dichroism (CD) spectra were obtained from the CD spectrometer (ChirascanTM-plus, Applied Photophysics, Ltd., Leatherhead, Surrey, UK) and temperature-regulated cells (25 °C) with 0.5 mm path and 1.0 nm bandwidth. The spectra were taken at pH 4.0, 5.0, and 6.0 in media mentioned above.

### In silico molecular docking

Since suitable templates for homology modeling were stored in the PDB, the amino acid sequences of mature cathepsin L and SnuCalCpIs were submitted to SWISS-Model (https://www.swissmodel.expasy.org) (Table S2). One template from the identification results was used as the guide based on its similarity and identity. The three-dimensional (3D) structures of SnuCalCpIs were built based on selected templates and visualized using Pymol software (version 2.5.2). The quality of the model was assessed by Ramachandran plots and QMEANDisCo Global (high numbers between 0 and 1 indicate high quality). Lastly, the *in sillico* molecular docking simulation of mature cathepsin L and SnuCalCpIs was performed by HDOCK (http://www.hdock.phys.hust.edu.cn) which provides both template-based modelling and *ab initio* free docking^20^.

## Results and discussion

### Molecular cloning and sequencing of SnuCalCpI genes

After 1^st^ strand cDNA synthesis, the eight DNA sequences of SnuCalCpI genes including SnuCalCpI02, SnuCalCpI03, SnuCalCpI08, SnuCalCpI12, SnuCalCpI14, SnuCalCpI15, SnuCalCpI16, and SnuCalCpI17 were amplified using specifically designed primers. Amplified SnuCalCpI gene fragments with sizes ranging from 320 to 380 bp were extracted, cleaned, and cloned into pET29b( +) vectors. Recombinant pET29b plasmids were transformed into competent *E. coli* DH5α cells. The DNA sequence of SnuCalCpI genes from recombinant plasmids was sequenced and verified by sequence alignment with cDNA sequences from *Calotropis procera* R. Br.

A multiple sequence alignment of SnuCalCpI genes was performed with a papain propeptide sequence (GenBank: AAB02650) and a human cathepsin L propeptide sequence (GenBank: AAH12612.1) to investigate sequence similarities and conserved regions (Fig. S2). The results showed that SnuCalCpIs consist of three different α-helices with helix-hinge-helix motifs commonly found in cathepsin L propeptides and papain propeptides^21^. In particular, the highly conserved regions, 'ERFNIN (EXXXRXXXFXXNXXXIXXXN)' motif which is located in the long α-helix II and constitutes a major part of the propeptide, and 'GNFD (GXNXFXD)' motif which is present at the kink between the β-sheet and the short α-helix III hindering the exposure of the interdomain cleft, among SnuCalCpIs indicate that they belong to a papain-like cysteine protease family^22^. The presence of the ERFNIN-GNFD motif also suggests that SnuCalCpIs can be classified as Inhibitor I29 according to MEROPS, which runs through the substrate-binding site and blocks substrate access. As such, SnuCalCpIs potentially inhibits the activity of human cathepsin L^12,23,24^.

### Expression of SnuCalCpIs in the *E. coli* system and purification

The recombinant plasmid for each SnuCalCpI gene was transformed into *E. coli* BL21(DE3) for protein expression. SDS-PAGE was used to analyze the solubility of the proteins for further study. The SDS-PAGE results in Fig. S1 displayed that water-soluble SnuCalCpI02, SnuCalCpI03, SnuCalCpI12, SnuCalCpI15, and SnuCalCpI16 were expressed at a high level. On the other hand, SnuCalCpI08, SnuCalCpI14, and SnuCalCpI17 could not be expressed under the selected conditions. Thus SnuCalCpI08, SnuCalCpI14, and SnuCalCpI17 were excluded in the subsequent experiments. Expressed proteins were purified by Ni–NTA affinity chromatography, and their molecular weights ranging from 13 to 16 kDa were confirmed by SDS-PAGE analysis (Fig. S3). It is not clear why overexpression in the *E. coli* system did not occur. However, it may be possible to achieve overexpression if we optimize expression conditions, e.g., codon usage, *E. coli* strain, temperature, and inducer concentration^25^.

### Inhibitory activity against papain and cathepsin L

**Figure 1:**
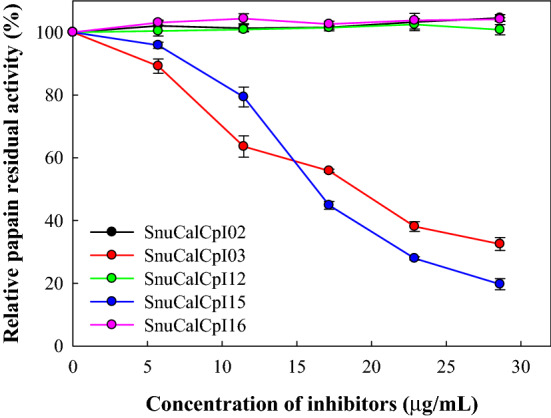
Inhibitory activity of SnuCalCpIs against papain.

Proteases, such as actinidain, caricain, cathepsin L, and ficain belong to the papain family C1 and are part of the clan CA cysteine proteases. They have zymogens and mature structures similar to those of papain and generally possess Cys25 and His159 residues in the catalytic dyad, and Gln19 and Asn175 residues in the active site that form the oxyanion hole and orientate the imidazole ring of the catalytic His. Papain was used as a representative enzyme of clan CA due to its well-known mature structure for understanding the inhibitory activity of SnuCalCpIs against papain-like cysteine protease. As a result, only SnuCalCpI03 and SnuCalCpI15 inhibited papain activity, whereas SnuCalCpI02, SnuCalCpI12, and SnuCalCpI16 did not show any inhibition against papain even though they have common conserved regions (Fig. 1). Therefore, we assumed that SnuCalCpI03 and SnuCalCpI15 would be effective inhibitors against papain-like cysteine proteases and consequently expected them to inhibit cathepsin L.

**Figure 2:**
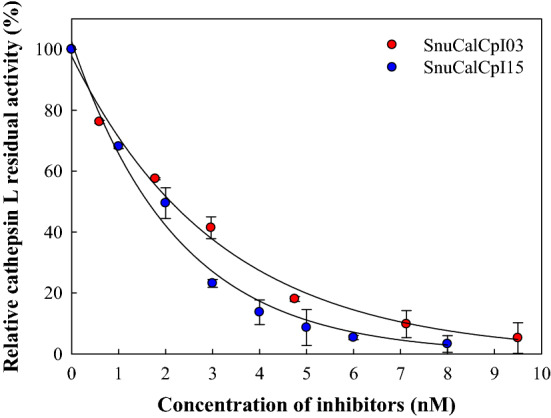
Inhibitory activity of SnuCalCpIs against human cathepsin L.

Inhibitory activity of SnuCalCpIs against cathepsin L was confirmed and compared by obtaining half-maximal inhibitory concentration (IC_50_) values. As shown in the result against papain, only SnuCalCpI03 and SnuCalCpI15 inhibited the activity of cathepsin L (Fig. 2). IC_50_ values of 2.1 nM and 1.6 nM were determined from SnuCalCpI03 and SnuCalCpI15, respectively, by plotting fitted curves of residual activity of cathepsin L. Their inhibition efficiency implies that the structures are more suitable for cathepsin L than papain.

### Types of cathepsin L inhibition

**Figure 3:**
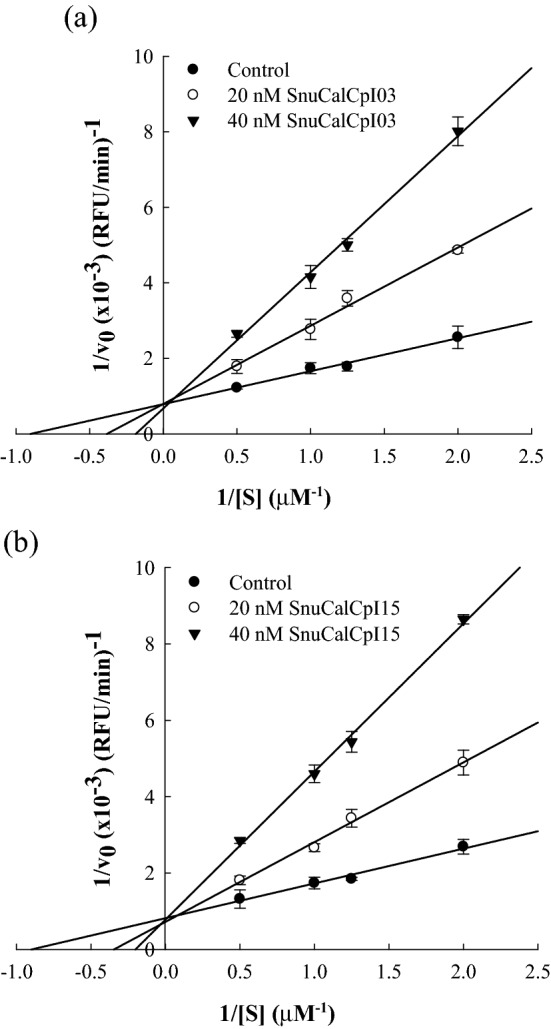
The Lineweaver–Burk plot for the determination of inhibition type (**a**) SnuCalCpI03 and (**b**) SnuCalCpI15.

A kinetic study of SnuCalCpI inhibition on cathepsin L was conducted to identify the mode of inhibition. A double reciprocal Lineweaver–Burk plot, widely used for enzyme kinetics, was adapted to elucidate the inhibition characteristics of SnuCalCpI03 and SnuCalCpI15. In the presence of SnuCalCpI03 and SnuCalCpI15, the slopes of the straight lines in the double reciprocal plot increased with increasing concentrations of SnuCalCpIs. *K*_m_ values at the x-intercept increased, but *V*_max_ values at the y-intercept did not change with the slope (Fig. 3). The concentrations of SnuCalCpIs affected both the horizontal axis and slope of the Lineweaver–Burk plot, indicating that they are competitive inhibitors.

The peptide inhibitors compete with the substrate for binding at the active site of cathepsin L, and consequently inactivate cathepsin L through reversible and non-covalent interactions. The weak binding of the peptide inhibitor formed from the non-covalent interactions may be desirable for specific enzyme targets as it has a higher selectivity and lower off-target toxicity than covalent inhibitors^22^. Specifically, reversible competitive peptide inhibitors recognize specific amino acid residues on the enzyme surface and active site through hydrogen bonding, hydrophobic, electrostatic, and van der Waals interaction^26^. Therefore, the inhibitor can interact selectively with a specific enzyme. On the other hand, some irreversible covalent inhibitors occasionally form a covalent bond with reactive molecules, e.g., cysteine and serine, even when they are not located in the active site of the target enzyme, resulting in immunological response or cell damage^27^.

### Thermal and pH stability

**Figure 4:**
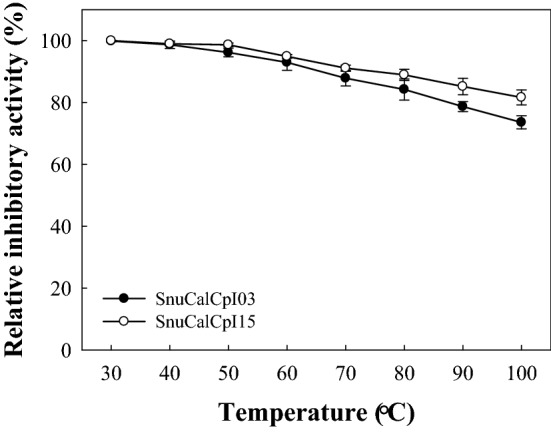
Thermal stability of SnuCalCpI03 (●) and SnuCalCpI15 (○).

To investigate their thermal stability, SnuCalCpI03 and SnuCalCpI15 were incubated at 30–100 °C for 30 min and cooled at 4 °C. Both inhibitors were stable and recovered more than 90% of their original inhibitory activity between 30 and 70 °C (Fig. 4). However, the residual inhibitory activity was reduced to 70% after incubation at 80–100 °C. These results imply that the thermal unfolding of the inhibitors was partial and reversible due to their intra-helical salt bridges or simple and compact tertiary structure. However, the lack of disulfide bonds might have decreased their thermal stability.

**Figure 5:**
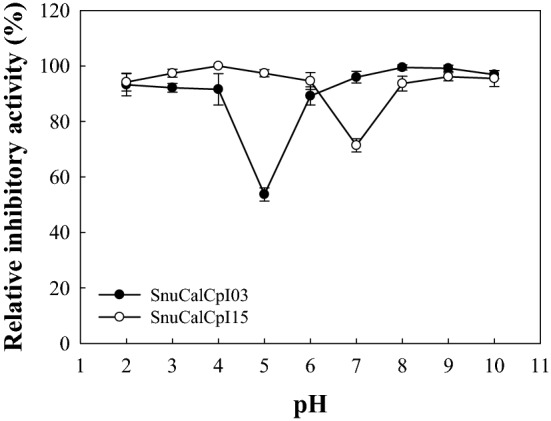
pH stability of SnuCalCpI03 (●) and SnuCalCpI15 (○).

**Figure 6:**
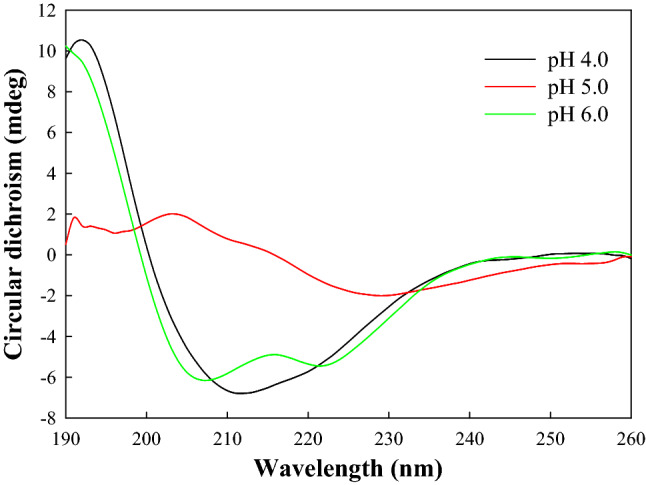
Far-UV CD spectra of SnuCalCpI03 at pH 4.0, 5.0, and 6.0^13^.

The pH stability profile showed that SnuCalCpI03 and SnuCalCpI15 are stable over the pH range from 2.0 and 10.0, except for 5.0 and 7.0, respectively, by maintaining more than 90% of its original inhibitory activity (Fig. 5). The negatively or positively charged structures of the peptide in acidic and alkaline conditions could be refolded into native structures because it remained in a molten globule state. However, a significant loss of inhibitory activity was found near the isoelectric point (pI) of 5.2 and 6.9 for SnuCalCpI03 and SnuCalCpI15, respectively. Therefore, it was concluded that they could not be restored after they precipitate at the pI. Although it seems that these peptide inhibitors are composed of three α-helices and one β-sheet, which are not considered sophisticated, there are several crucial interactions to maintain the globular fold: (i) hydrophobic core formation between α-helixes; (ii) salt bridges between α-helix and β-sheet strand; and (iii) a hydrophobic minicore between α-helix and cathepsin L^28^. The far-UV CD spectra of SnuCalCpI03 over the pH range from 4.0 to 6.0 supported the above mentioned loss of interactions and inhibitory activity near the pI (Fig. 6). At pH 6.0, an intense positive band at 193 nm, an intense negative band at 208 nm, and a shoulder at 218 nm suggest a high proportion of amino acid residues integrated into α-helical secondary structures. The CD spectrum at pH 4.0 was similar to that at pH 6.0 except that there was no shoulder at 218 nm due to the secondary structure alteration induced by acidification. In contrast, the CD spectrum at pH 5.0 did not show any distinct band intensities over the wavelength range from 190 to 230 nm unlike those at pH 4.0 and 6.0.

From the stability test results and far-UV CD spectra, it was expected that SnuCalCpI03 and SnuCalCpI15, which are stable over a wide range of temperatures and pHs, except at the pI, could be stored and applied in diverse environments.

### In silico molecular docking analysis

*In sillico* molecular docking simulation of mature cathepsin L and SnuCalCpIs was conducted to analyze why some SnuCalCpIs have inhibitory activity against cathepsin L, and others have not, even though they have ERFNIN and GNFD motifs which are important elements for inhibiting activity of papain-like cysteine proteases. We performed molecular docking analysis with template-based prediction because activation of the proenzyme does not cause critical conformational changes for the mature cysteine protease and its propeptide^28^. In addition, the quantitative docking scores including HDOCK score and ligand rmsd (Å) from template-based modeling were compared with those of *ab initio* free docking. Before proceeding with the sumulations, the quality of the template-based models generated by SWISS-MODEL was analyzed and compared. The analysis with QMEANDisCo and favored regions of Ramachandran plots demonstrated good quality of all models except SnuCalCpI16 (Table 1).

**Table 1:** Quality assessment scores of modeled structures.

Model	GMQE	QMEANDisCo global	Ramachandran favored (%)
SnuCalCpI02	0.49	0.57 ± 0.09	94.68
SnuCalCpI03	0.50	0.65 ± 0.09	87.23
SnuCalCpI12	0.49	0.69 ± 0.09	90.24
SnuCalCpI15	0.46	0.69 ± 0.10	97.50
SnuCalCpI16	0.08	0.26 ± 0.12	84.85

**Figure 7:**
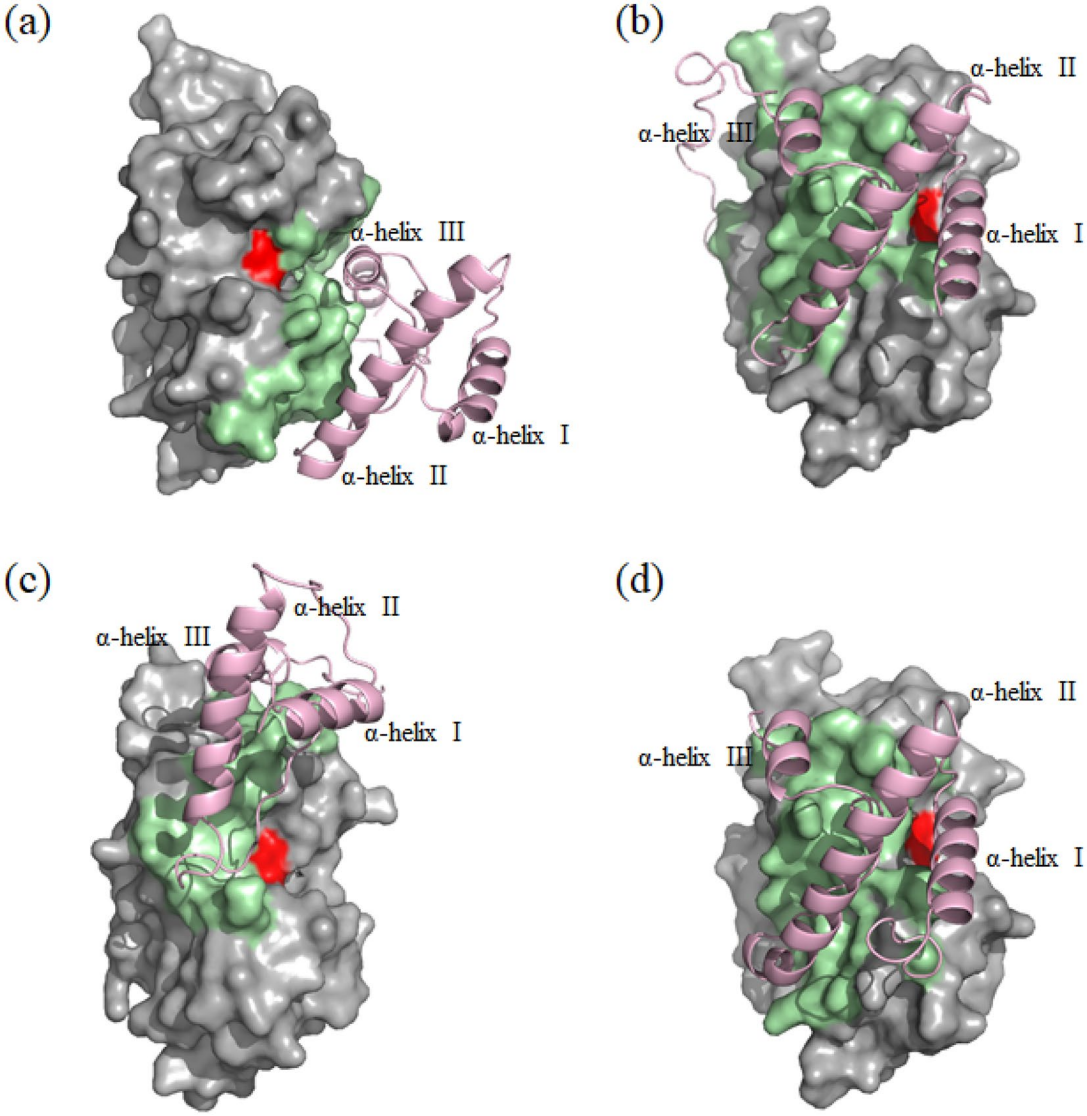
Protein–protein docking simulation of cathepsin L and SnuCalCpIs. (**a**) cathepsin L-SnuCalCpI02 complex, (**b**) cathepsin L-SnuCalCpI03 complex, (**c**) cathepsin L-SnuCalCpI12 complex, and (**d**) cathepsin L-SnuCalCpI15 complex. The enzyme and inhibitor were represented in gray and lightpink, respectively. The catalytic dyad (Cys25 and His163) and interface residues were illustrated in red and palegreen, respectively. The protein–protein complex was visualized using Pymol software (version 2.5.2).

The best prediction models out of ten obtained from HDOCK were shown in Fig. 7 and docking scores of them were enumerated in Table 2. The simulation models showed that only SnuCalCpI03 and SnuCalCpI15 blocked the active site cleft of cathepsin L, which verifies inhibitory activity of them, although the quantitative docking score and ligand rmsd (Å) did not show significant difference among the predicted protein–protein complexes. The SnuCalCpI03 and cathepsin L interactions occured in three propeptide binding loops of cathepsin L: surface formed by the Thr 14-Cys 21, Phe 143-Gly 149, and Trp 189-Gly 194. The presence of α-helix III is considered not essential for inhibitory activity but plays an important role in maintaining cathepsin L-SnuCalCpI03 complex. On the other hand, α-helix II and adjacent amino acid residues are essential and stabilize the structure by forming a hairpin structure and allow it to interact with the propeptide binding loop through hydrogen bonds and hydrophobic interactions. Thereby, α-helix III can interact with amino acid residues in the vicinity of catalytic dyad (Cys25 and His163) and block substrate access. The inhibition mode of SnuCalCpI15 was similar to that of SnuCalCpI03, but it was slightly different because it lacked C-terminal region contributing little to overall inhibition. To cross-check simulation results from template-based models, *ab initio* free docking was conducted. However, it was impossible to compare the simulation results with those of sequence-based docking because the protein–protein interaction occurred at a great distance from the catalytic dyad.

**Table 2:** Quantitative docking scores for cathepsin L-SnuCalCpI complex.

Model	Structure-based docking		Sequence-based docking	
	HDOCK score	RMSD (Å)	HDOCK score	RMSD (Å)
SnuCalCpI02	− 250.97	50.57	− 255.83	175.39
SnuCalCpI03	− 196.35	71.93	− 206.32	44.47
SnuCalCpI12	− 202.45	40.93	− 285.44	198.54
SnuCalCpI15	− 236.99	80.44	− 212.34	32.52

## Conclusions

This study evaluated the inhibitory activity of SnuCalCpIs against cathepsin L, which plays a crucial role in cancer cell migration through ECM degradation. The inhibition mechanism was investigated by adopting a bioinformatics approach. Only SnuCalCpI03 and SnuCalCpI15 out of five expressed SnuCalCpIs could inhibit cathepsin L activity by blocking substrate access to the active site of cathepsin L, although all the SnuCalCpIs have highly conserved domains and form α-helix structures. *In silico* protein–protein docking simulations, especially structure-based modeling, can be useful to validate experimental or predicted results.

## Supplementary Information

Supplementary Information 1.

Supplementary Information 2.

Supplementary Information 3.

Supplementary Information 4.

Supplementary Information 5.
