# Supplementary material for: Characterization and molecular docking study of cathepsin L inhibitory peptides (SnuCalCpIs) from Calotropis procera R. Br
Source: Sci Rep. 2022 Apr 6;12:5825. doi: 10.1038/s41598-022-09854-x (PMC8986768; doi:10.1038/s41598-022-09854-x)
Supplement: Supplementary file 1 — Supplementary Information 1. [file 41598_2022_9854_MOESM1_ESM.docx]

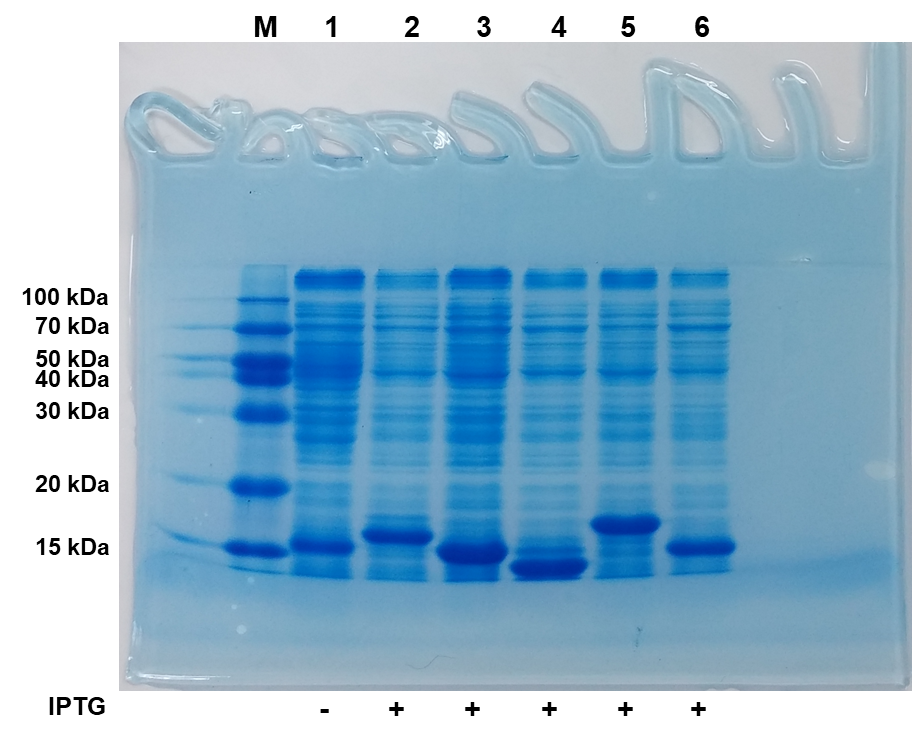
Fig. S1. SDS-PAGE analysis of recombinant SnuCalCpIs expressed in *E. coli* system. lane M, protein size marker; lane 1, control; lane 2, SnuCalCpI02; lane 3, SnuCalCpI03; lane 4, SnuCalCpI12; lane 5, SnuCalCpI15; lane 6, SnuCalCpI16.
