# Supplementary material for: Characterization and molecular docking study of cathepsin L inhibitory peptides (SnuCalCpIs) from Calotropis procera R. Br
Source: Sci Rep. 2022 Apr 6;12:5825. doi: 10.1038/s41598-022-09854-x (PMC8986768; doi:10.1038/s41598-022-09854-x)
Supplement: Supplementary file 2 — Supplementary Information 2. [file 41598_2022_9854_MOESM2_ESM.docx]

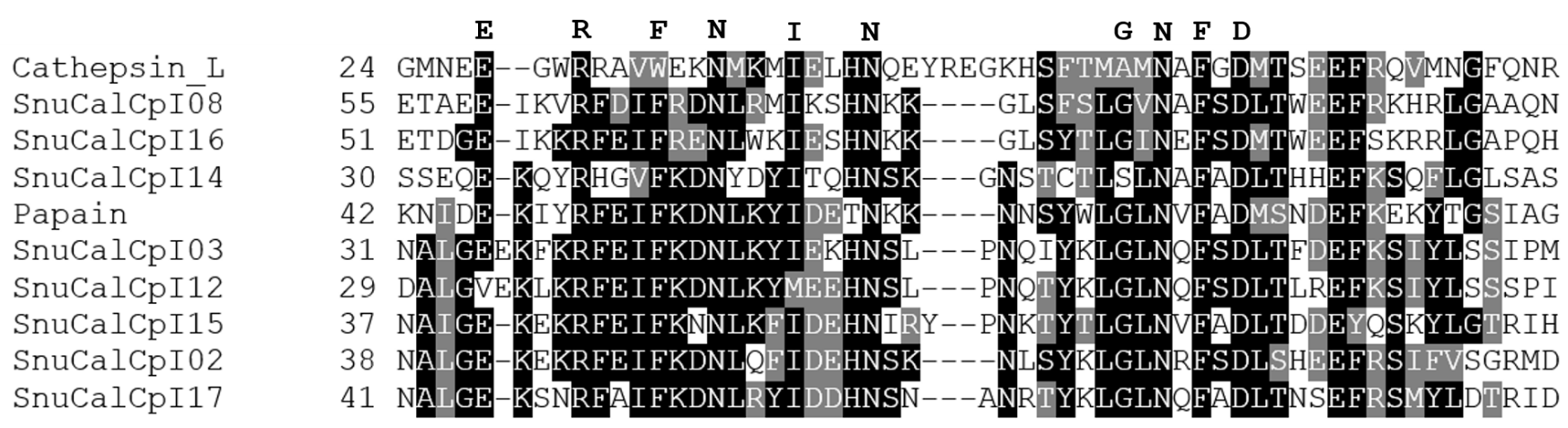


Fig. S2. Multiple protein sequence alignment of SnuCalCpIs with papain propeptide and human cathepsin L propeptide by Clustal Omega.
