# Supplementary material for: Characterization and molecular docking study of cathepsin L inhibitory peptides (SnuCalCpIs) from Calotropis procera R. Br
Source: Sci Rep. 2022 Apr 6;12:5825. doi: 10.1038/s41598-022-09854-x (PMC8986768; doi:10.1038/s41598-022-09854-x)
Supplement: Supplementary file 3 — Supplementary Information 3. [file 41598_2022_9854_MOESM3_ESM.docx]

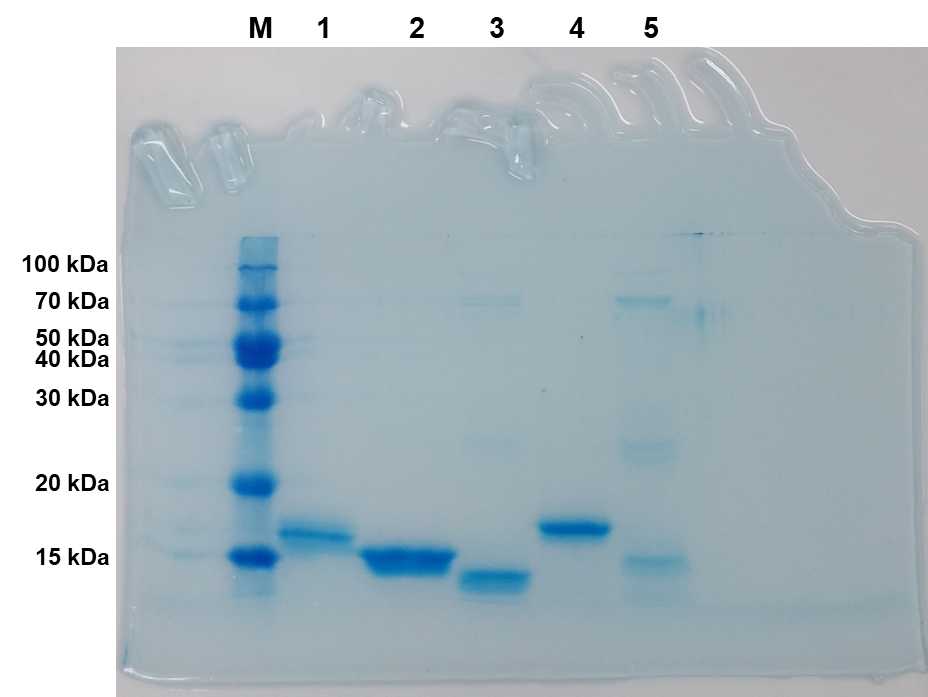
Fig. S3. SDS-PAGE analysis of affinity-purified recombinant SnuCalCpIs. lane M, protein size marker; lane 1, SnuCalCpI02; lane 2, SnuCalCpI03; lane 3, SnuCalCpI12; lane 4, SnuCalCpI15; lane 5, SnuCalCpI16.
