# Supplementary material for: Characterization and molecular docking study of cathepsin L inhibitory peptides (SnuCalCpIs) from Calotropis procera R. Br
Source: Sci Rep. 2022 Apr 6;12:5825. doi: 10.1038/s41598-022-09854-x (PMC8986768; doi:10.1038/s41598-022-09854-x)
Supplement: Supplementary file 4 — Supplementary Information 4. [file 41598_2022_9854_MOESM4_ESM.docx]

**Table S1** Oligonucleotide primers used in cloning of cysteine protease inhibitors.

| **Primer name** | **Sequence (5’ to 3’)** |
| --- | --- |
| SnuCalCpI02_F | AAGGAGATATACATATGTTAGACATGTCCATTATCAGT |
| SnuCalCpI02_R | GGTGGTGGTGCTCGAGATCATCACCAGCATTAAAAGA |
| SnuCalCpI03_F | AAGGAGATATACATATGAAAATCATATCCATTGCCGAT |
| SnuCalCpI03_R | GGTGGTGGTGCTCGAGGTTAAGGTCAACTTCAGAAAA |
| SnuCalCpI08_F | AAGGAGATATACATATGGTTGACGACGGATCATCAG |
| SnuCalCpI08_R | GGTGGTGGTGCTCGAGGACAACGTTGGTTAGCTTG |
| SnuCalCpI12_F | AAGGAGATATACATATGATTGCCGATGAATTAGTCCG |
| SnuCalCpI12_R | GGTGGTGGTGCTCGAGAGAAAGGTTATAGTTAACTTGG |
| SnuCalCpI14_F | AAGGAGATATACATATGTCATTTTCATCTTCTTCTTCTT |
| SnuCalCpI14_R | GGTGGTGGTGCTCGAGATCAAAATCATCAAAGACATCT |
| SnuCalCpI15_F | AAGGAGATATACATATGATCATCACTACTAGCCTCC |
| SnuCalCpI15_R | GGTGGTGGTGCTCGAGGCTTTCAGATCCAACTTTGT |
| SnuCalCpI16_F | AAGGAGATATACATATGGACCGTTCATCATTCTCCG |
| SnuCalCpI16_R | GGTGGTGGTGCTCGAGAGCAACGTTGTTGAGCTTTA |
| SnuCalCpI17_F | AAGGAGATATACATATGTCTGAGATCACGTCGGTTA |
| SnuCalCpI17_R | GGTGGTGGTGCTCGAGATTATCTTCGGCTTTAGGAAG |
