# Supplementary material for: Characterization and molecular docking study of cathepsin L inhibitory peptides (SnuCalCpIs) from Calotropis procera R. Br
Source: Sci Rep. 2022 Apr 6;12:5825. doi: 10.1038/s41598-022-09854-x (PMC8986768; doi:10.1038/s41598-022-09854-x)
Supplement: Supplementary file 5 — Supplementary Information 5. [file 41598_2022_9854_MOESM5_ESM.docx]

**Table S2** Amino acid sequences of expressed SnuCalCpIs and mature cathepsin L.

| Peptide | Sequence |
| --- | --- |
| SnuCalCpI02 | MLDMSIISYDNDHGQMVRSDDEVRSLYESWLVKHGKAYNALGEKEKRFEIFKDNLQFIDEHNSKNLSYKLGLNRFSDLSHEEFRSIFVSGRMDRKARLMKGKVGDRYSFNAGDDLEHHHHHH |
| SnuCalCpI03 | MISIADESVWRTEEEVMAIYEEWIVKHGKSYNALGEEKFKRFEIFKDNLKYIEKHNSLPNQIYKLGLNQFSDLTFDEFKSIYLSSIPMDTSLSESKIDFSEVDLNFPLEHHHHHH |
| SnuCalCpI12 | MIADELVRRTDEEVMSIYEEWMVEYRKSYDALGVEKLKRFEIFKDNLKYMEEHNSLPNQTYKLGLNQFSDLTLREFKSIYLSSSPIDTLLDESEIDFSYFPQVNYNLSLEHHHHHH |
| SnuCalCpI15 | MIITTSLHNSQNKLVWRTNDEVISLFEEWLVKHRKVYNAIGEKEKRFEIFKNNLKFIDEHNIRYPNKTYTLGLNVFADLTDDEYQSKYLGTRIHPKRKYFASHSSDDDEYLHKVGSESLEHHHHHH |
| SnuCalCpI16 | MATLLSFRVEYLVAEQCCGAPNLRLLNSSHVISENSLMPRVYESPFLLWDSIFHKFSLNISNLFLISPSVSYFFPYLRAKLANERTWRLSPTTSRKEASSSRRRSATTCLIGFSSENDERLEHHHHHH |
| Mature cathepsin L | APRSVDWREKGYVTPVKNQGQCGSCWAFSATGALEGQMFRKTGRLISLSEQNLVDCSGPQGNEGCNGGLMDYAFQYVQDNGGLDSEESYPYEATEESCKYNPKYSVANDTGFVDIPKQEKALMKAVATVGPISVAIDAGHESFLFYKEGIYFEPDCSSEDMDHGVLVVGYGFESTESDNNKYWLVKNSWGEEWGMGGYVKMAKDRRNHCGIASAASYPTV |
